# Investigating the link between *Helicobacter pylori* infection and idiopathic thrombocytopenic purpura a case–control study

**DOI:** 10.1097/MS9.0000000000004331

**Published:** 2025-11-25

**Authors:** Inayat Ullah, Syed Hamza Arshad, Mazhar Khalil, Maryam Iftikhar, Abid Ullah, Abdur Rahman Qazi, Arif Ullah, Zarak Qureshi, Irfan Ullah, Sardar Noman Qayyum, Maria Qadri, Rafiullah Hotak

**Affiliations:** a Department of Internal Medicine, Lady Reading Hospital, Peshawar, Pakistan; b Department of Medicine, Wrightington Wigan and Leigh NHS Trust, United Kingdom; c Department of Internal Medicine, Hayatabad Medical Complex, Peshawar, Pakistan; d Department of Medicine, Khyber Teaching Hospital, Peshawar, Pakistan; e Department of Medicine, DHQ Teaching Hospital, Bannu, Pakistan; f Department of Medicine, Lady Reading Hospital Peshawar, Peshawar, Pakistan; g Department of Medicine, Blackpool Teaching Hospitals NHS Foundation Trust, United Kingdom; h Department of Medicine, Bacha Khan Medical College, Mardan, Pakistan; i Department of Medicine, Jinnah Sindh Medical University, Karachi, Pakistan; j Nangarhar University, Faculty of Medicine, Nangarhar, Afghanistan

**Keywords:** autoimmune thrombocytopenia, case–control study, Helicobacter pylori, immune thrombocytopenic purpura, molecular mimicry

## Abstract

**Background:**

Immune thrombocytopenic purpura (ITP) is an autoimmune disorder marked by isolated thrombocytopenia. Recent studies suggest a link between *Helicobacter pylori* (*H. pylori*) infection and ITP, possibly through molecular mimicry and immune-mediated mechanisms. This study aimed to investigate the association between *H. pylori* infection and ITP in a Pakistani population where both conditions are prevalent.

**Methods:**

A case–control study was conducted from May 2022 to June 2024 at a tertiary care hospital in Pakistan. A total of 141 participants were enrolled, including 46 patients with primary ITP and 95 age- and sex-matched controls without thrombocytopenia. All subjects were tested for *H. pylori* using stool antigen tests, urea breath tests, and confirmatory biopsies where applicable. Logistic regression was used to assess the association while adjusting for potential confounders.

**Results:**

*H. pylori* infection was present in 39.1% of ITP patients compared to 17.9% of controls (*P* = 0.0026). The crude odds ratio (OR) was 2.96 (95% CI: 1.36–6.46), and the adjusted OR was 3.18 (95% CI: 1.42–7.13, *P* = 0.005) after controlling for age, gender, smoking, and socioeconomic status. Other variables were not significantly associated with ITP.

**Conclusion:**

A significant association between *H. pylori* infection and ITP was observed, supporting the need for routine screening in ITP patients, especially in endemic areas. Further studies should evaluate the platelet response after *H. pylori* eradication and assess the role of bacterial strain variability.

## Introduction

Immune thrombocytopenic purpura (ITP), a disorder of platelets, is an autoimmune condition causing destruction of the platelets due to the generation of antibodies against the platelet’s antigen^[^1^]^. This disease is determined by excluding other diseases; its association with a bacterium is still under investigation. ITP is solely a decrease in platelet count, i.e., a count of less than 1 50 000 cells/mm^3,^ and can cause problems such as being asymptomatic to causing life-threatening bleeding episodes^[^2^]^.

**HIGHLIGHTS**

- A case–control study conducted in Pakistan shows a significant association between *Helicobacter pylori* infection and immune thrombocytopenic purpura (ITP).
- *H. pylori*-positive individuals had nearly three times higher odds of having ITP compared to those without infection.
- The association remained significant after adjusting for confounding factors such as age, gender, smoking status, and socioeconomic background.
- This is one of the first studies from the region to provide local evidence supporting the role of *H. pylori* in ITP pathogenesis.
- Findings support the potential benefit of *H. pylori* screening and eradication as part of ITP management, particularly in endemic areas.

The main mechanism that occurs in this disease is that platelet production is impaired because the IgG antibodies bind to glycoproteins on the platelet’s membrane, causing platelets to sequester and then be phagocytosed within the spleen^[^1^]^. The disease is immune-mediated, so when the cause of the disease is not identifiable, it is suggestive of primary ITP, but when the thrombocytopenia is due to any identifiable cause, then it is secondary ITP. Although this disease has severe outcomes, the diagnosis of the disease is still challenging as it requires excluding all the other causes of low platelet count^[^1^]^.

Although there are many causes contributing to this, *Helicobacter pylori*, a gram-negative, flagellated bacterium existing as helical, rod, or curved shapes, is notoriously associated with this disease. There are certain hypotheses, but the main accepted hypothesis is that of molecular mimicry. The bacterium has a cytotoxin-associated gene A (Cag-A) against which antibodies are formed, which cross-react with platelets’ antigens, contributing to their destruction and engulfment^[^3^]^. Another antigen expressed by this bacterium, called the Lewis antigen, also has a similar structure to that of platelets’ antigen, thus also causing cross-reactivity and platelet destruction^[^4^]^. Also, more efficient phagocytosis by monocytes during *H. pylori*’s infective period is also a contributing secondary ITP^[^5^]^. Certain human leukocyte antigen (HLA) types accounting for host genetic susceptibility, such as HLA-DRB111, 14, and HLA-DQB103, are also associated with increased risk of ITP associated with *H. pylori*, while HLA-DRB103 is less frequently associated with this disease^[^6^]^. The dysbiosis in intestinal flora has also been linked to the pathogenesis of ITP^[^7^]^.

Importantly, several studies have shown that eradication of *H. pylori* infection can lead to a significant improvement in platelet counts in ITP patients, suggesting that routine screening and eradication may not only help identify underlying causes but also serve as a therapeutic strategy in selected patients^[^8^] [^9,10^]^. Recent systematic reviews and meta-analyses confirm that eradication therapy is associated with platelet recovery in a subset of ITP patients, though responses vary across populations^[^11^]^. Large cohort- and registry-based studies and several clinical series have shown that patients with *H. pylori* infection who receive successful eradication therapy are more likely to experience platelet recovery than infected patients who do not receive eradication; conversely, lack of eradication is associated with a higher subsequent risk of developing severe thrombocytopenia in some cohorts^[^12,13^]^.

Systematic reviews and meta-analyses across geographic regions indicate that eradication therapy leads to a statistically significant overall increase in platelet counts and higher odds of response in many, but not all populations. Response rates vary by region and by study design, suggesting heterogeneity that may reflect differences in *H. pylori* strain prevalence (e.g., Cag-A-positive strains), host genetics, and baseline patient characteristics. These pooled analyses support the inclusion of *H. pylori* testing in the diagnostic workup of chronic ITP, where local prevalence is high, while recognizing that not all patients will respond^[^14^]^.

Mechanistically, recent reviews emphasize molecular mimicry (CagA and Lewis antigens), enhanced monocyte-mediated platelet phagocytosis during infection, and an emerging role for gut microbiome alterations in modulating immune responses in ITP. Newer work has begun to explore how regional strain variation and host HLA types may modify the likelihood of platelet recovery after eradication, which helps explain variability in clinical response across studies^[^15,16^]^.

This is little information on the possible correlation between *H. pylori* and ITP in Pakistan, where both infections are prevalent, especially in Khyber PakhtunKhwa (KPK). This study uses a case–control design at a tertiary care hospital to determine whether there is a statistically significant correlation between *H. pylori* infection and ITP. The results can enhance knowledge of the possible involvement of *H. pylori* in the etiology of ITP and look for the advantages of routine screening or eradication.

## Methodology

A prospective case–control study was conducted at a tertiary care hospital in Pakistan to investigate the association between *H. pylori* infection and ITP. The study was carried out over 2 years, from May 2022 to June 2024, at a tertiary care hospital. Ethical approval was obtained from a tertiary care hospital’s ethical review committee. The study followed international guidelines.

The sample size was calculated using OpenEpi.com, which yielded a sample size of 141 using exposure prevalence of 30% (based on previous studies)^[^14,17,18^]^ an expected odds ratio (OR) of 3, with a 95% confidence interval (CI) and 80% power. The control-to-case ratio was maintained at 1:2 to increase the statistical power.

Participants were recruited using a consecutive sampling approach, whereby all eligible patients presenting during the study period were enrolled. A total of 141 participants were enrolled and divided into two groups using a 1:2 case-to-control ratio. In the case group, 46 patients diagnosed with primary ITP were enrolled, while in the control group, 95 participants were enrolled from the same tertiary care hospital with no history of thrombocytopenia and a normal platelet count.

The inclusion criteria for the case group were: patients aged 18 or above and diagnosed with primary ITP as per the American Society of Hematology guidelines, such as a platelet count less than 100 × 10^9^/L, and exclusion of secondary causes of ITP (drugs, HCV, HIV)^[^19^]^. The exclusion criteria included patients with thrombocytopenia secondary to known causes, patients with recent use of antibiotics, and pregnancy or known immunodeficiency illnesses.

Informed consent was obtained from all individuals prior to their participation in our study. A detailed clinical evaluation was performed, including assessment of gastrointestinal (GI) symptoms, smoking history, socioeconomic background, and family history of GI disorders or malignancy. All participants were tested for *H. pylori* infection using stool antigen testing, serology antibody tests, urea breath test, and confirmatory gastric mucosal biopsy.

Data were analyzed using SPSS v27. Descriptive statistics were used to summarize the demographic and clinical variables. A chi-square was used to assess the association between *H. pylori* and ITP. ORs with 95% CIs were calculated to explore the strength of association. The study followed the STROCCS guidelines in reporting the findings^[^20^]^.

## Results

A total of 141 participants were enrolled, comprising 46 cases diagnosed with ITP, and 95 age- and sex-matched controls with no history of thrombocytopenia. The mean age in the ITP group was 39.5 ± 12.3 years, and 38.9 ± 11.5 years in the controls (*P* = 0.74, 95% CI for mean difference: −2.9 to 4.1). The age and gender distributions were similar in both groups, as shown in Table 1. Low socioeconomic status was reported by 63% of the ITP patients compared to 50.5% in controls (*P* = 0.18, OR = 1.65, 95% CI: 0.79–3.46). Smoking was reported by 41.3% of ITP patients and 32.6% of controls (*P* = 0.31, OR = 1.45, 95% CI: 0.70–2.99).

**Table 1:** Demographic characteristics of the study population

Characteristics	ITP patients (*n* = 46)	Controls (*n* = 95)
Mean age (± SD)	39.5 ± 12.3 years	38.9 ± 11.5 years
Age-group in years		
s18–30	14 (30.4%)	33 (34.7%)
31–45	20 (43.5%)	38 (40 %)
46–65	12 (26.1%)	24 (25.3%)
Gender		
Male	24 (52.2%)	46 (48.4%)
Female	22 (47.8%)	49 (51.6%)
Low socioeconomic	29 (63.0%)	48 (50.5%)
Smokers	19 (41.3 %)	31 (32.6%)

ITP was diagnosed among cases as per the guidelines of AHS, and later on confirmed by an independent hematologist. For *H. pylori* infection detection, the stool antigen test and urea breath test were used. Both were revealed to be positive in the participants deemed positive for *H. pylori*. In instances of discordant results, a gastric mucosal biopsy was performed, and biopsy findings were taken as the reference standard. This ensured validity and reduced the risk of misclassification bias.

Of the 46 ITP patients, 18 (39.1%) tested positive for *H. pylori*, compared to 17 (17.9%) among the controls. This difference was statistically significant by the Chi-square test (χ^2^ = 9.10, *P* = 0.0026). The crude OR was 2.96 (95% CI: 1.36–6.46), suggesting that *H. pylori*-positive individuals had nearly three times the odds of having ITP compared to those without infection.

After adjusting for age, gender, smoking status, and economic status, the adjusted OR was 3.18 (95% CI: 1.42–7.13, *P* = 0.005). Thus, *H. pylori* remained a significant predictor of ITP after adjusting for confounders. However, other covariates were not statistically significant. The Hosmer–Lemeshow test for model fit turned out to be non-significant (*P* = 0.58), which is suggestive of good model calibration. While the CI indicates some variability, the consistency across statistical methods strengthens the validity of the findings, as shown in Table 2. Gastrointestinal complaints were reported by 24 ITP patients compared to 20 patients in the control group (*P* = 0.001, OR = 4.11, 95% CI: 1.85–9.11). Family history of GI disorders and malignancies was also reportedly high in ITP patients (*P* = 0.004, OR = 3.27, 95% CI: 1.46–7.33).

**Table 2:** Association between *H. pylori* infection and ITP

*H. pylori* sstatus	ITP patients (*n* = 46)	Controls (*n* = 95)	*P*-value	Odds ratio (95% CI)
Positive	18	17	0.0026	2.96 (1.36–6.46)
Negative	28	78		

## Discussion

The findings of this case–control study depict a strong and statistically significant association of *H. pylori* with ITP. Patients with *H. pylori* infection had approximately three times more likelihood of having ITP compared to controls (adjusted OR: 3.18, 95% CI: 1.42–7.13). These findings are consistent with the early findings in the literature found regionally or globally.

A meta-analysis conducted in Japan and Italy showed the same association of *H. pylori* and ITP, and platelet count improved significantly in patients who were treated completely for *H. pylori*^[^10^]^. Another study found that patients without *H. pylori* eradication therapy had higher odds of developing ITP (adjusted OR 1.76; 95% CI 1.16–2.68)^[^21^]^. The biological evidence of this is further supported by previous literature showing that the Cag-A-positive strains of *H. pylori* are associated with a higher incidence of ITP, showing a more aggressive behavior of this strain in patients^[^22^]^. Therefore, complete eradication of *H. pylori* should be done mainly in CagA-positive patients as a treatment option for ITP. It is also important to take environmental and genetic influences into account, as evidenced by the variable response rate to *H. pylori* treatment noted across various regions such as Italy, Japan, Spain, and the United States^[^18^]^. The goal should be to eliminate these factors to maximize the therapeutic response^[^23^]^. Vonoprazan-based triple therapy and non-bismuth quadruple therapy are considered the best treatment regimen, as evidenced by the literature for complete eradication^[^24^]^.

Although the effectiveness of *H. pylori* eradication in raising the platelet count is controversial, it has still shown beneficial outcomes^[^25^]^. The results to investigate the potential benefit of eradicating *H. pylori* was not assessed in our study. Previous literature shows that certain patients of ITP showed improvement as their platelet count returned to near normal after successful eradication of *H. pylori* infection^[^8^]^. There is more likelihood of platelet’s recovery after the successful treatment for *H. pylori* as evidenced by a clinical trial^[^21^]^. However, for the eradication therapy, the response rate varies significantly from 30% to 70%, which depends mainly on the diagnostic method used and the population being investigated. Therefore, to assess the impact of successful eradication of *H. pylori* infection on ITP, strict diagnostic criteria along with long-term follow-up need to be established to minimize this variability^[^20^]^.

The additional factors, such as smoking and socioeconomic status, also have a slight role in the causation of ITP, but the significance of this is quite low. Smoking might act as an immunomodulator in the causation of this disease, along with socioeconomic status, which might exacerbate the progression of this disease. A study conducted in the Japanese population shows that the risk of *H. pylori* infection decreases with increasing socioeconomic status (OR = 0.67, 95% CI [0.53–0.85] for low socioeconomic status and OR = 0.43, [0.34–0.54] for moderate to high socioeconomic status)^[^26^]^.

The relationship between *H. pylori* and ITP has been described by various mechanisms, one of which is mainly acceptable, called Molecular mimicry, which is when antibodies are formed against the bacterial antigen, but they cross-react with glycoproteins on the platelets’ surface. It is proposed that this cross-reactivity mainly results from the immune system mistakenly targeting platelets due to the similar antigenic structure of certain *H. pylori* strains and platelets’ surface antigen^[^27^]^. The immune-mediated response is higher with strains positive for Cag-A^[^28^]^. Moreover, in genetically susceptible individuals, certain strains of *H. pylori* expressing Lewis antigen can also attach to platelets and start producing autoantibodies^[^29^]^. Other findings show that *H. pylori* might enhance the phagocytosis of platelets by monocytes, thus accounting for thrombocytopenia^[^30^]^.

Additionally, the process of megakaryopoiesis is also affected when inflammatory cytokines are produced due to *H. pylori*-mediated chronic inflammation.^[^31^]^ Immune dysregulation occurs as the bacterium also produces interleukins, interferon gamma, and tumor necrosis factor, which further exacerbate the condition^[^32^]^. The growth, maturation, and differentiation of megakaryocytes are suppressed due to cytokines; thus, the count of platelets produced is decreased^[^33^]^. Variation in mechanisms is due to factors such as genetic susceptibility of the host, environmental effects, and different virulence of *H. pylori* strains, and investigations are done to find the precise mechanism^[^34^]^.

Although the strength of the association may vary depending on the population under study and the diagnostic method used, the calculated OR of 3 demonstrates a significant association. *H. pylori* infection remained a single significant factor for ITP even after adjusting for other variables such as smoking, socioeconomic status, after a multivariable logistic regression was done. This highlights the importance of screening patients affected with ITP for *H. pylori* while considering the diagnosis of low platelet count and treatment options, especially in areas where *H. pylori* infections are prevalent.

In countries like Pakistan, such study findings have an important role related to public health because of the high prevalence of *H. pylori*, mainly due to overcrowding, poor diet and sanitation, smoking, and limited access to healthcare. Thus, considering *H. pylori* infection as one of the causes of low platelet count may provide a cost-effective and noninvasive way to improve the platelet count and lessen the burden of disease. In order to stop the spread of *H. pylori*, the implications of better hand hygiene and awareness regarding *H. pylori* also have a significant role.

### Limitations of the Study

Although the study suggests strong evidence, it has certain limitations. The multivariable analysis was done taking into account the smoking status and socioeconomic condition of the participants, but it lacked other factors such as nutritional status, other infections, and sanitation. Additionally, potential misclassification bias may have occurred due to the reliance on stool antigen and urea breath tests for *H. pylori* detection, as no single diagnostic method is 100% accurate. Also, the different strains of *H. pylori* were not stratified, such as Cag-A-positive, Lewis’s antigen positive strains, etc., which may have made the analysis more accurate.

### Public Health Implications

The findings of this study underscore the potential role of *H. pylori* screening in regions where both infection and ITP are prevalent. Early identification and treatment of *H. pylori* could not only improve patient outcomes by reducing the risk of chronic thrombocytopenia but may also prove to be cost-effective by preventing unnecessary investigations and long-term therapies. In resource-limited settings such as Pakistan, integrating *H. pylori* screening into routine clinical practice for patients with unexplained thrombocytopenia could represent a practical public health strategy. However, large-scale cost-effectiveness studies are needed to confirm this benefit before widespread implementation.

## Conclusion

This study depicts a significant association between ITP and the role of *H. pylori* in its pathogenesis. The OR suggests that the prevalence of ITP was three times higher in ITP patients infected with *H. pylori* as compared to healthy individuals. While this finding supports a possible contributory role of *H. pylori* in ITP pathogenesis, the cross-sectional design does not establish causality. The multifactorial nature of this disease is proven by taking into account the socioeconomic condition and smoking status of the participants. The study also highlights the importance of screening for *H. pylori* in endemic areas where the infection is prevalent. In order to confirm the results and improve the recommended treatment plans, a larger sample size, post *H. pylori* eradication, platelet response, and various *H. pylori*-specific strain analysis is needed for future research.

## Data Availability

The dataset used and analyzed during the current study are available from the corresponding author, Rafiullah Hotak, upon reasonable request.

## Research registration unique identifying number (UIN)

The study has been registered on the Open Science Framework (OSF) and is available at: https://doi.org/10.17605/OSF.IO/ZEKVU.
